# Evaluating Automatic Body Orientation Detection for Indoor Location from Skeleton Tracking Data to Detect Socially Occupied Spaces Using the Kinect v2, Azure Kinect and Zed 2i †

**DOI:** 10.3390/s22103798

**Published:** 2022-05-17

**Authors:** Violeta Ana Luz Sosa-León, Angela Schwering

**Affiliations:** Spatial Intelligence Lab, Institute for Geoinformatics, University of Münster, 48149 Muenster, Germany; schwering@uni-muenster.de

**Keywords:** RGB-D sensors, human motion modelling, F-Formation, Kinect v2, Azure Kinect, Zed 2i, socially occupied space

## Abstract

Analysing the dynamics in social interactions in indoor spaces entails evaluating spatial–temporal variables from the event, such as location and time. Additionally, social interactions include invisible spaces that we unconsciously acknowledge due to social constraints, e.g., space between people having a conversation with each other. Nevertheless, current sensor arrays focus on detecting the physically occupied spaces from social interactions, i.e., areas inhabited by physically measurable objects. Our goal is to detect the socially occupied spaces, i.e., spaces not physically occupied by subjects and objects but inhabited by the interaction they sustain. We evaluate the social representation of the space structure between two or more active participants, so-called F-Formation for small gatherings. We propose calculating body orientation and location from skeleton joint data sets by integrating depth cameras. The body orientation is derived by integrating the shoulders and spine joint data with head/face rotation data and spatial–temporal information from trajectories. From the physically occupied measurements, we can detect socially occupied spaces. In our user study implementing the system, we compared the capabilities and skeleton tracking datasets from three depth camera sensors, the Kinect v2, Azure Kinect, and Zed 2i. We collected 32 walking patterns for individual and dyad configurations and evaluated the system’s accuracy regarding the intended and socially accepted orientations. Experimental results show accuracy above 90% for the Kinect v2, 96% for the Azure Kinect, and 89% for the Zed 2i for assessing socially relevant body orientation. Our algorithm contributes to the anonymous and automated assessment of socially occupied spaces. The depth sensor system is promising in detecting more complex social structures. These findings impact research areas that study group interactions within complex indoor settings.

## 1. Introduction

While studying how people interact in space, alone or with a companion, the first approximation is to identify variables describing movement and measure them. Specific parameters are straightforward to determine as they can be physically detected in space; for example, the location of people involved in a conversation and their distances can be assessed as properties of physically occupied space. Other aspects describing interactional processes are invisible to the eyes, but still, people inside or outside the group well understand and respect them [1]. While sensors are able to measure the physical properties of people and their location, to date, it is still a challenge to detect their interaction automatically; however, it exists due to social accords in a socially occupied space that is not physically discernible [2]. Sociology studies gatherings of people to identify different roles such as leadership [3], with raised interest to detect them in entertainment to take pictures [4], to help to arrange displays in an interaction encouraging way [5], to improve the communication and design in virtual reality [6], and in computer vision to improve the way robots approach individuals [7,8]. To date, research on identifying interactions among people or between people and their environment often relies on manual observation techniques based on video recording [9]. Other approaches for static scenes analyse videos to detect groups of people automatically by extracting social cues [10]. The distinction between physical and non-physical space is one key unsolved challenge in the automatic interpretation of interactional spaces.

**Figure 1:**
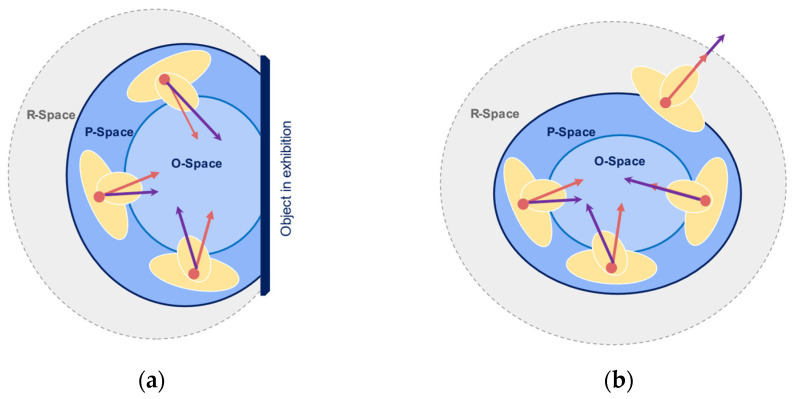
The illustration of the F-Formation model and its three interactional areas are O, P, and R spaces. In (**a**) group–object interaction. In (**b**) group–members interaction.

Currently, sensor-based systems focus on spaces physically appropriated by a human body or an object, so-called physically occupied spaces. On the contrary, we aim to detect the socially occupied space, i.e., space occupied not physically by people. Social models such as facing formations, so-called F-Formations, represent this occupancy that occurs due to a social agreement. F-Formations are present when “two or more individuals maintain a spatial and orientational interaction in which the space between them is one with equal, direct and exclusive access” [11]. The model comprises three areas: O-space, the inner transactional space; P-Space, the narrow zone immediate to the O-Space, and R-Space, which protects the system and serves as a transactional space for the participants, as shown in Figure 1. The interactional space is the area in which the interchange occurs, existing between the bodies involved in the exchange [12]. After analysing people’s bodies and participation during an interaction, it is then possible to conclude the interactional space. Sociologists have physically distinguished social interaction models by implementing direct observation, interviews, and analysing videos [13,14], concluding that body orientation is crucial in encouraging participation from all members [13]. Nevertheless, the difficulty in detecting these social spaces rises with the number of people, i.e., the size of the gathering; thus, automating the physical features’ measurement to describe them is vital. Defining the socially occupied space requires discerning where people stand and their body’s direction to detect their interactional space placement.

Moreover, spatial–temporal information is needed to rate the level of engagement in a conversation and describe the encounter’s physical dynamics. Tracking technologies such as Bluetooth and Wi-Fi are used to extract position and movement, helping to detect encounter dynamics [14,15]. However, they lack information about bodily signs to identify individuals’ interaction with the milieu or everybody else.

Our study concentrates on extracting the data needed to interpret the socially occupied space and defining a methodology to obtain it from different sensor devices. We select a set of depth cameras, with infrared and stereoscopic technology, the Kinect v2, Azure Kinect, and Zed 2i, to tell people’s position, body orientation, and viewing direction, which are central in explaining group interaction. Then, we implement the F-Formations model, a social model, by translating the sensors’ measurements into an interpretation of the socially occupied space for small size and highly focussed gathering interactions. From the results, we evaluate which device suits better our use case. Our approach does not rely on video storage or trackers’ placement, unlike other methods. The emphasis is to assess the different sensor’s output data in detecting body orientations. We collect data for eight body orientations in four different walking patterns for each depth camera. The designed algorithm uses the shoulders and spine skeleton joints information collected, together with the trajectories’ temporal information, to calculate the body angle. The accuracy evaluation consists of the following methods: evaluating whether the automatic body orientation falls into the correct category with a body orientation category classification, followed by a category deviation analysis, and finally, versus an acceptable social orientation range. Our experiment results show accuracy above 90% for both the Kinect v2 and Zed 2i and 95% for the Azure Kinect for assessing the body angle in the experiment setup, with the different depth sensors’ accuracy varying in specific areas for side, back and diagonal body orientations and location to the device. In this paper, our contributions are:

- We compare three different depth sensors to evaluate the use of the skeleton data generated by their depth maps and calculate the body orientation from the skeleton data and assess the sensors’ accuracy by analysing the link between location and intended direction. Additionally, we analyse the advantages and disadvantages per device in determining the body orientation.
- We can conclude the spatial extent of the personal interactional space from the body’s location and orientation. The focus of attention that intersects allows us to identify people in group interaction and the resulting interactional space.
- We create a system to collect information from physically occupied spaces, analysing the relevant information to interpret socially occupied spaces.

The structure of the paper is as follows: Section 2 introduces related literature to track people and discover groups in indoor spaces. Section 3 depicts the system configuration and our skeleton data processing approach. Section 4 and Section 5 describe the experiment setup, the system evaluation, and the discussion of the results for each format and device. Finally, we present in Section 6 our conclusions and future work.

## 2. Related Work

**Social interaction.** In analysing human social behaviour, interactions can evolve from a single individual to an increasing number of participants. Social structures such as groups are defined as a social unit with more than one individual and a clear membership that sustain a continuous interaction. Complementary gatherings represent an interaction often in public spaces, defined as a set of two or more co-present individuals sharing a temporal interaction [16]. As the number of individuals decreases, the situation possesses a different level of interaction: large encounters with thirty-one to N participants happen in semi-public and public spaces such as concerts, whereas medium gatherings from seven to thirty participants arise in meetings and classrooms. The larger the number of participants in a gathering, a lower level of common focus exists, whereas lesser members showcase solid social interaction and group belonging [17]. Small gatherings from two to six participants imply a common-focused or jointly focused interaction, where people are involved in a mutual activity [18] encompassing conversational groups, which can be studied within the F-Formation model.

**Figure 2:**
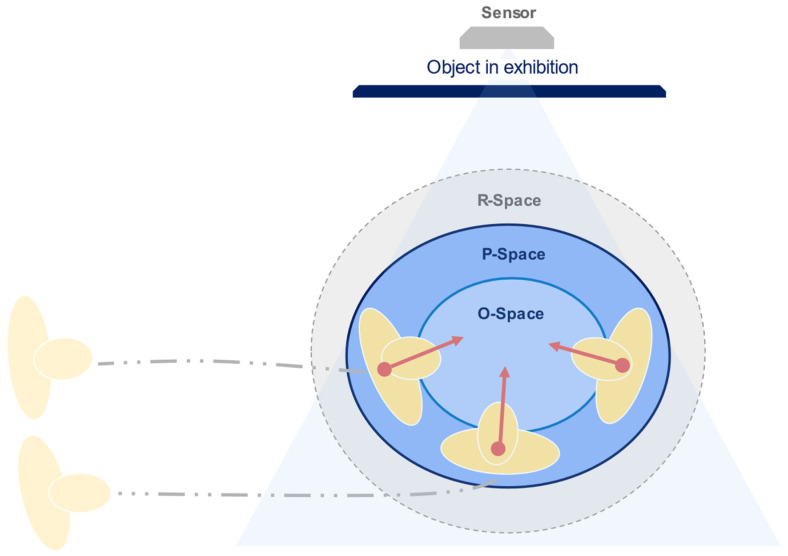
A set of individuals join a third member and construct the interactional space. The position and body orientation establish physically which space is socially occupied. Spatial–temporal variables such as position over time indicate the dynamics of interaction.

F-Formations exist when “two or more individuals maintain a spatial and orientational interaction in which the space between them is one with equal, direct and exclusive access” [11]. The model comprises three areas: O-space, the inner transactional space in which the focus of attention is present; P-Space, the narrow zone immediate to the O-Space where individuals position themselves; and the R-Space, which protects the system and serves as an entry and exit point for the participants, enclosed by the bodies orientation [19]. The detection of these areas relies on the definition of the *orientational transaction* to assess the intersection of focus of attention in an interactional zone, for which Kendon integrated the concept of social proxemics. Hall defined four physical areas from human observations in social situations: intimate, personal, social and public zones [13]. The interpersonal distance in social zones ranges from 1.20 to 2.10 m. These zones are integrated into the F-Formation model to address its extension and the area in which interactants, and their focus of attention exists as illustrated in Figure 2. The field of view in which the attention spans, is represented by a cone with origin in the frontal body, with a aperture value of around 120°; inside this cone, the inner cone in which humans sustain attention during interaction ranges between 30° and 60° degrees, the so-called gaze area [20]. The different stages of attention can assess the focus during interaction during trajectories [21,22]. During the capture stage, attention is unfocused, and individuals’ actions rely on scanning and approaching elements in the environment. Narrow attention arises in the focus stage, where the attention is captured for fewer than three seconds in a single object. Finally, when the attention is deep for more than five seconds, reaching the engagement stage, the bodies are static in a position, and senses are concentrated on reading, discussing, or recalling content, generating a social experience in which the interactional spaces are constructed by the bodies participating in the interchange of information [23]. Our research focuses on small gatherings in indoor spaces, particularly museum exhibitions, by analysing the position and an approximation of the body orientation from static social encounters. The goal is to identify the components of the socially occupied space for highly engaged moments during an interaction.

**Human behaviour tracking approaches.** Different studies have been implemented to evaluate individuals’ position in interaction in closed spaces [15,24]. For our analysis, these human tracking technologies can be divided into devices with and without physical contact with the user. The first category is unobtrusive because its installation is in the surroundings. For example, Wi-Fi and Bluetooth technologies help identify device interaction and location. However, its utility is limited to positional variables. It does not directly assess body data to analyse social interactions, giving partial information about people’s spatial and body arrangement. They need additional data such as video recordings or manual records [25]. LiDAR cameras in museums have similar limitations for measuring social interactions with exhibitions, requiring significant processing tasks to get precise trajectories, deriving information mainly about highly concurred areas [26]. Finally, computer vision techniques to identify human traffic rely on RGB cameras to detect people’s bodies and derive their trajectories, incurring privacy concerns and challenges such as occlusion and distortion. The second category is obtrusive as it is installed in peoples’ bodies in the form of trackers and markers, interfering with their activities’ natural behaviour, especially when users are required to activate beacons to confirm their locations [27]. So far, these technologies focus mainly on spatial data, offering only proximity information to identify groups according to their shared space.

Nevertheless, due to the richness in human interactions, trajectories need to be complemented with relevant data to characterise the interactional space and offer more context for the sociological analysis of models such as F-Formations [9]. The description of social features in group interaction has been studied in museum visits using forms and manual observation, implying expensive and lengthy analysis [23]. Additional techniques involve using cameras to design traditional and interactive displays in closed spaces, reducing the socially occupied space to an area to be physically occupied [28]. Existing computer vision methods use video datasets such as SALSA and Babble and identify attention, proximity, and head orientation to analyse participants in a conversation with the analysis of bodies from video recordings, highlighting the difficulty of the analysis of head rotation as a result of a low-resolution video [29,30]. Other similar studies rely on virtual environments to recreate social dynamics [31]. These approaches focus solely on detecting conversation’s physically occupied space, ignoring the surrounding socially occupied space dynamics that led to these groups’ construction.

**Depth cameras for human interaction.** Several depth camera models are available outside the industrial market, such as the Orbbec and the Intel RealSense models used for 3D image extraction, depth map reconstruction and gait analysis [32,33,34]. Each device provides different software solutions to process scene information, such as semantic segmentation, object detection and skeleton tracking, open to the public or with a fee, as shown in Table 1. Cameras with the skeleton tracking functionality ready to be used without cost for researchers and practitioners in their studies include the Microsoft Kinect series and the Zed 2i. 

Approaches using commercial depth cameras include the Kinect v2 camera in an egocentric perspective in robots for conversational participation and events, limiting the analysis to static scenarios evaluating only the physically occupied space by interacting with the artificial participants [35,36]. However, this use demonstrates their great potential in acquiring trajectory and relevant social features due to the processed skeleton data, easiness of installation, and low costs without storing video data from the scene, allowing researchers to exploit these data to extract human behaviour [37,38,39]. Additional depth cameras available for the public include the Azure Kinect, the successor of the Kinect v2, mainly used for industry and healthcare with promising human activity detection [40]. Studies comparing both devices are limited to joint detection accuracy for medical monitoring, static scenarios, or physical training that does not reflect the natural movement of the body in large trajectories [41,42]. An alternative depth camera to the Time-of-Flight technology from the Microsoft devices is the Zed 2i from Stereolabs, which relies on stereoscopic technology to gather depth information and extract body joints. Nevertheless, only selected studies are available to evaluate the depth map accuracy from the previous model [32,43], and research on the skeleton joints accuracy model is scarce.

This study intends to extend these prior studies by integrating social signals, trajectories, and human behaviour. Social signals describe a set of behavioural attitudes from social intelligence present during interaction [44]. The posture and gesture category highlights the relevance of low-level social features: distance, aperture, and body orientation to assess interaction [6,45]. We use depth cameras as a hybrid technology for tracking individuals and collecting body data during trajectories to automate detecting the invisible space in interaction. To assess which depth camera technology and model is more suitable for detecting socially occupied spaces, we compare the skeleton tracking data generated by two infrared-based and one stereoscopic-based depth camera.

## 3. System Design

**Figure 3:**
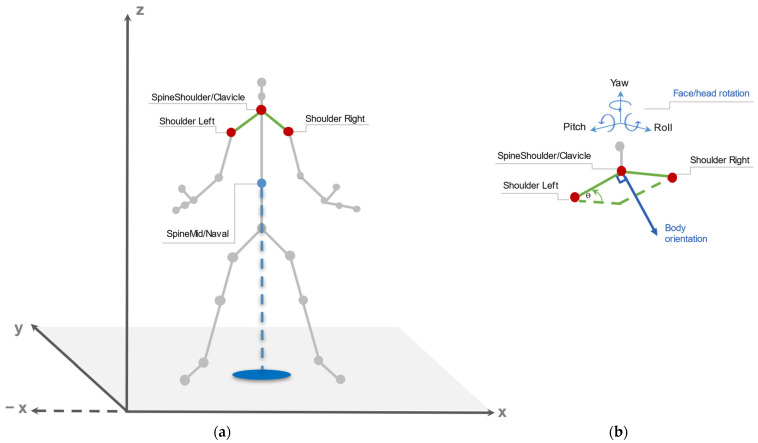
Real−world coordinate system with the skeleton extracted from the depth cameras. On (**a**) the selected skeleton joints are in red, with the positional skeleton joint in blue. On (**b**) the selected upper skeleton joints used to calculate body orientation.

We propose employing depth sensors cameras to extract the body orientation using skeleton tracking data joints, with a series of evaluations assessing the efficacy of detecting F-Formations exploiting spatial–temporal and body cues data. Our methodology comprises five steps: a set of data collection experiments, a coordinates transformation and social cues processing, the estimation of the body angle orientation and evaluation, and finally we test our findings with a group detection algorithm. We process the shoulder left, right, and centre joints, as described in Figure 3, to calculate body angle orientation and the spine joint’s coordinates as the position for each skeleton dataset generated collected per device: two infrared-based and one stereoscopic-based technology.

### 3.1. Depth Sensor Cameras

We selected three depth sensor cameras that are reachable to end-users in terms of the price, market availability to the public, capability to generate skeleton tracking data, easiness of installation and running within different environments. From Microsoft, the Kinect v2 and the Azure Kinect offer end-users a device with capabilities ranging from games to industrial use using Time-of-Flight (TOF) technology. Alternatively, Stereolabs with the Zed 2i offers a stereoscopic camera whose size and configuration make it a good option for developers in robotics and industry.

#### 3.1.1. Kinect v2

Microsoft launched the Kinect v2 in 2016 as an accessory for the XBOX console to track a body’s movements for video games. The device extracts the scene depth information by processing the incoming light using an infrared and RGB-D video. The device detects 25 body joints per skeleton for up to six bodies using a set of decision tree-based algorithms with no native information for the head/face elements. The Microsoft Kinect Software Development Kit (SDK) allows to access the device, basic tutorials and depict the camera status, limited to Windows operating systems versions higher than 8. Additionally, the libraries can be implemented in WPF C# projects to access its functionalities [46], adding others, such as a complementary face elements detection, including eyes, mouth and head from the Microsoft.Kinect.Face library.

#### 3.1.2. Azure Kinect

The next generation of Kinect devices came in 2020 with the introduction of the Azure Kinect. The Azure focuses on industrial warehousing, robotics, and health applications compared to the previous generation [37]. The Azure camera uses the highest hardware specification requirements from the three devices. The depth camera implements an amplitude-modulated continuous Wave Time-of-Flight principle, casting illumination in the near-IR spectrum to record the light travelled. The skeleton tracking feature includes 32 body joints including face elements for up to four bodies, employing a neural network algorithm to derive the skeleton bodies from the depth map. Users can access the camera functionalities with the SDK and libraries written in C++ and C# on Windows and Linux operative systems, possibly connecting to Azure Cognitive Services for other processes.

#### 3.1.3. Zed 2i

Stereolabs Zed 2i depth camera has been available in the market since 2021 and is based on stereoscopic technology by using two 4Mpx sensors, calculating the displacement of the pixels between the left and the right images captured. The body tracking is based on a neural network algorithm to detect body joints present on both sides, and it merges the information with the depth and positional tracking model, producing 34 body joints, including face components. The camera requires configuring CUDA and a Zed-specific development environment to access the SDK functionalities, which work on Windows and Linux operative systems [47].

**Figure 4:**
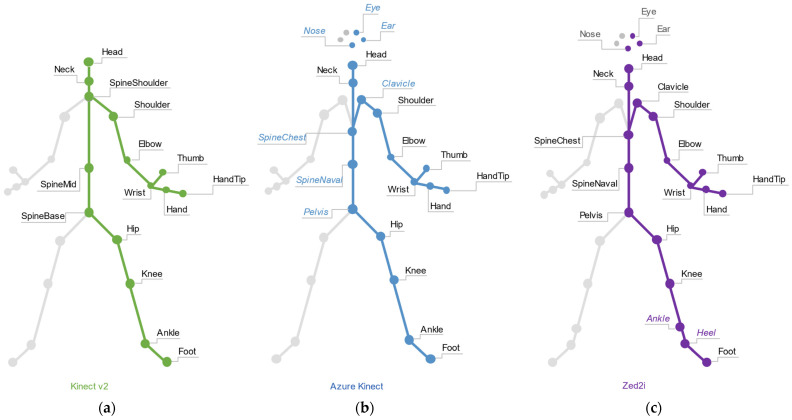
Skeleton joints map per device. (**a**) Kinect v2, (**b**) Azure Kinect and (**c**) Zed 2i. Greys areas indicate a left-right joint correspondence. Italic joints indicate differences between the devices.

The body skeleton joints structure and the coordinate system for each camera are shown in detail in Figure 4, with a summary and technical comparison in Table 2. The Azure Kinect and the Zed 2i have the highest number of joints, including face and spine ones with slightly different names.

### 3.2. Depth Cameras Coordinates’ Transformation

**Figure 5:**
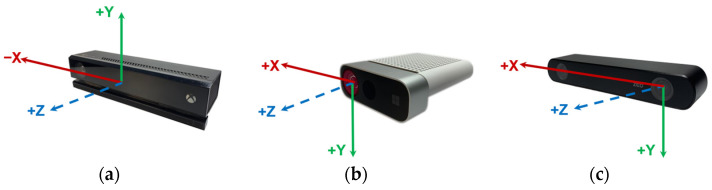
Depth camera sensors with their coordinate system: (**a**) Kinect v2, (**b**) Azure Kinect, and (**c**) Zed 2i. The Zed 2i has six different coordinate systems.

We transform the device’s coordinates into the physical space. The devices generate data relative to their positions in their own coordinate system, with x representing the positive or negative horizontal distance, y the height, and z the frontal distance, as shown in Figure 5.

The coordinates system origin $x_{o},\;y_{o}$ are set to the device position, calculating the transformed coordinates $x_{t},y_{t}$, with a translation angle *θ*, and the camera’s original set of coordinates $\left[x_{k},y_{k},z_{k}\right]$ for each skeleton joint. We apply a matrix product transformation described in Equation (1):

(1) $$\left[x_{t},y_{t}\right]=\left[\begin{array}{ccc} \cos\theta & -\sin\theta & x_{o}\\ \sin\theta & \cos\theta & y_{o}\\ 0 & 0 & 1 \end{array}\right]\left[\begin{array}{c} x_{k}\\ y_{k}\\ 1 \end{array}\right]$$

### 3.3. Processing Social Signals

**Figure 6:**
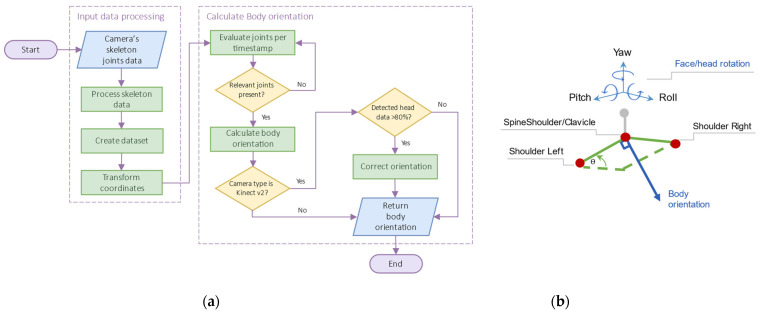
On (**a**) Methodology to extract body orientation from the skeleton data collected. On (**b**) illustration of upper joints and head rotation data usage to calculate the body orientation.

Humans regularly recognise the direction in which the body is oriented by identifying the head orientation and the upper and lower limbs and reviewing if they are placed left or right. Following this reasoning, the body orientation is calculated from the skeleton joints tracked by each device. The data generated by each sensor in the form of JSON files contain information about every set of joint coordinates, as illustrated in Figure 3. We collect each skeleton data every 200 milliseconds, using the upper limbs as an indicator of the focus of attention to later calculate the body angle. The main methodology is illustrated in Figure 6. The task starts by *processing the input data*. We receive the collected skeleton data in a JSON file, which is analysed and organised by timestamp, creating a dataset with all relevant information for the algorithm, such as body identification, skeleton joints, timestamp, body location, camera, and experiment identifier. Next, the program proceeds with the coordinates’ transformation for the body location and each skeleton joint.

Once the data is processed, the next step is to *calculate the body orientation*. For each position $p_{i}$, we review the availability of the interested upper joints to proceed with the angle calculations. This allows us to warranty the use of complete skeleton data sets as they can be incomplete every other timestamp. The algorithm evaluates if the relevant skeleton joints are present on each timestamp, selecting the upper joints shoulder left and right for all devices, shoulder centre and head orientation detected for the Kinect v2, and clavicle for the Azure Kinect and the Zed 2i. We use Equation (2) to calculate the body angle orientation after applying the corresponding coordinate transformation using Equation (1):

(2) $$\theta_{body\_angle}=\arctan\left(\frac{x_{t}}{y_{t}}\right)*\;\frac{180}{\pi }$$

To assess if the vector perpendicular to the orientation vector should rotate clockwise or counter-clockwise, we evaluate the position of each shoulder joint relative to the sensor’s orientation. If the left shoulder joint in the position $sl_{x}$ is larger than the right shoulder joint position $sr_{x}$, the body is looking in the direction of the sensor. This can be observed by plotting the shoulder line and the joint’s position as illustrated in Figure 6b.

Lastly, if the camera is the Kinect v2 and the head elements are available for more than 80% of the sample, we apply an additional correction to the orientation using a full 180° rotation as described in Algorithm 1. While Azure Kinect and Zed 2i include information on the face and head joints in detecting skeletal joins related to body orientation, Kinect v2 processes skeletal joints and face and head joints separately. As a result, front and back orientation are often confused, and the device has a strong tendency always to assume a front orientation, even if people are oriented backwards. To make all three approaches comparable, we integrate the face and head joins explicitly into the body orientation processing in the case of Kinect v2, where information from the head is processed. This operation potentially improves assessing the body orientation towards the camera as its absence suggests a non-frontal orientation [2]. On the other hand, the Azure Kinect and Zed 2i offer the face and head joints detected by the algorithm, but as they are already processed to evaluate the joints’ left-right correspondence internally, they are not included in the body orientation correction to avoid overfitting. In the end, the calculated angle $\theta_{pi}$ is returned. Algorithm 1 describes the mentioned process:
**Algorithm 1** Body angle calculation**Input:** A dataset *N* with skeleton joints in the form (x,y) per timestamp**Output:** Body orientation angle $\theta_{srsl}$
   **for**$p_{i}$ in *N*:  **if** shoulder_joint_pair:   apply_coordinates_transformation$\left(sl_{xy},\;sr_{xy}\right)$
    $\theta_{pi}=\tan^{-1}\left(\frac{x_{t}}{y_{t}}\right)*\left(\frac{180^{{}^{\circ}}}{\pi }\right)$
   **if** $sr_{x}<sl_{x}:$
     $\theta_{pi}=\theta_{pi}-90{}^{\circ}$
    **else:**     $\theta_{pi}=\theta_{pi}+90{}^{\circ}$
  **else:**    $\theta_{pi}=1$
  correction_level = analyze_head_data_availability($p_{i}$)   **if** camera is Kinect_v2 **and** correction_level > 80%:   apply_orientation_correction ($\theta_{pi})$
   return $\theta_{pi}$
**end**

### 3.4. An F-Formation Social Model for Group Detection

We integrate the F-Formations model for processing the physically occupied spaces to detect socially occupied spaces. From Kendon’s theory of F-Formations [11], the attributes to be extracted from the physical space are related to proximity, spatial–temporal data such as position and time, and focus of attention. We extract Kendo’s model attributes and proceed to identify shared stops among subjects in the conducted experiments. Hall defined proxemics with a range of 0.5 to 1.5 m distance between bodies as the personal space for two or more individuals coexisting in a continuous lapse of time [13] and a common focus of attention, to a person or an object, as the intersection of field of views [48].

We develop a group detection algorithm that uses the bodies’ trajectories and the calculated body angle on every timestamp as described in Algorithm 2.
**Algorithm 2** Group detection**Input:** A dataset *N* with skeleton joints in the form (x,y) per timestamp   an integer *stops_expected* with the number of assigned stops    an integer *groups_expected* representing the number of assigned group locations**Output:** A dataset *class_group* with group membership, and stop locations body_identification=spatemp_stop_kmeans(N, stops_expected)**for**$b_{i}$ in body_indentification:   trajectory_stops = spatemp_stop$\left(b_{i},\;t,r\right)$
shared_stops=intersection_stops(trajectory_stops)class_group=spatemp_stop_kmeans_time(shared_stops, groups_expected)**return** class_group

Firstly, we assign a body identifier by applying a spatial K-Means supervised classification algorithm with *stops_expected* parameter, the expected number of members in the scene. Next, we process the trajectory for each skeleton body *b_i_*, and detect individual stopping moments by evaluating the spine joints’ temporal and spatial proximity within a radius *r* and stop time *t*, generating the *trajectory_stops*. With the individual bodies’ long stop detected, we proceed to extract the individual stops intersected, assessing their coexistence in a maximum of 1.5 m personal space. Once the *shared_stops* in the trajectory are extracted, we apply a temporal K-Means supervised classification algorithm to evaluate group temporality with the parameter *groups_expected*, obtaining each *class_group* to assign group membership to each skeleton body. The focus of attention and its intersection is visualised by integrating the body angle calculation results and generating the body’s field of view. With this information, is possible to draw the F-Formation model components and thus the socially occupied space during the participants’ interaction.

In brief, our system employs three different depth sensor cameras to collect skeleton data during trajectories, from which we can extract the position and orientation of every participant. Once the skeleton joints data is collected and available, we apply a coordinates transformation to have a unified coordinate system as each device possesses its own. Secondly, we calculate the body orientation angle based on three skeleton joints: shoulders (left and right) and shoulder centre. Then, we proceed with an angle correction for the Kinect v2 to adjust the results to the same level as the other devices for a fairer skeleton joint algorithm comparison. To identify the most reliable camera for detecting socially occupied spaces, we assess the results with a set of performance evaluations. Finally, to probe the use of this approach, the attributes extracted from the physically occupied space are exploited to identify when group members are sharing an interactional space and focus of attention, thus the construction of an F-Formation.

## 4. Body Orientation Angle Evaluation

This section describes the experimental setup for two different configurations and shows the evaluation results, demonstrating the sensors’ body orientation accuracy.

### 4.1. Data and Software Availability

The data collected during these studies are available at https://osf.io/xhwgm/ (accessed on 15 March 2022). The tutorials and code to create a new interface for all devices can be found at https://github.com/violetasdev/bodytrackingdepth_course (accessed on 15 March 2022). For the Azure Kinect we implement a modified version of k4.net, the final version is available at https://github.com/violetasdev/k4a.net (accessed on 15 March 2022). The Kinect v2 body orientation plots can be reproduced at https://osf.io/ghz79/ (accessed on 15 March 2022). 

### 4.2. Experiment Setup

The sensors are positioned in an isolated 4.0 m × 9.0 m area, over a 2.0 m vertical truss with a height of 1.83 m, pointing towards a white wall. In the separated free floor area, coloured feet are placed every 1.2 m to cover each sensor’s field of view and guide participants to draw different walking patterns. Regarding the equipment configuration, the Kinect v2 sensor is connected to an Intel Core i7-10 laptop, with 16 GB of DDR4 RAM and a NVIDIA GeForce RTX 3070 Super Max-Q graphics card. The Zed 2i is connected to a laptop with Intel Core i9-11, 32 GB of RAM, and an NVIDIA GeForce RTX 3080 graphics card. The Azure Kinect is connected to an Intel Core i7-10 laptop with 16 GB of RAM and an NVIDIA GeForce GTX 1650 graphics card.

We produce the JSON files containing the skeleton joints data from three different coded solutions. The libraries implemented are in C# for the Kinect v2 and the Azure Kinect devices and in Python for the Zed 2i. A video camera records the computer screens for each trajectory to further review specific timestamps from the scene in search of external factors affecting the experiment. The skeleton data are collected every 200 milliseconds and the exact same scene setup for all devices, with the same starting time and bodies entering the scene simultaneously for the Azure Kinect and the Zed 2i. The resulting Kinect v2 skeleton data is taken from our previous data collection with the same configuration [2]. We use the narrow view configuration for the Azure Kinect for the skeleton tracking algorithm recommended by the fabricant due to the performance results [49]. For the Zed 2i, we select the COORDINATE_SYSTEM_IMAGE as the coordinate system to match all devices [50].

### 4.3. Participants’ Description

Two participants perform the oriented-walking patterns in a single and dyad configuration. First, one female with a height of 1.73 m follows each assigned pattern and body orientation for a total of 32 samples. Secondly, a dyad (a group composed of two participants) with a female and a male with a similar height ranging between 1.73 m and 1.81 m concludes an equal task while keeping a side-to-side configuration.

### 4.4. Walking Trajectories and Body Orientations Definition

**Figure 7:**
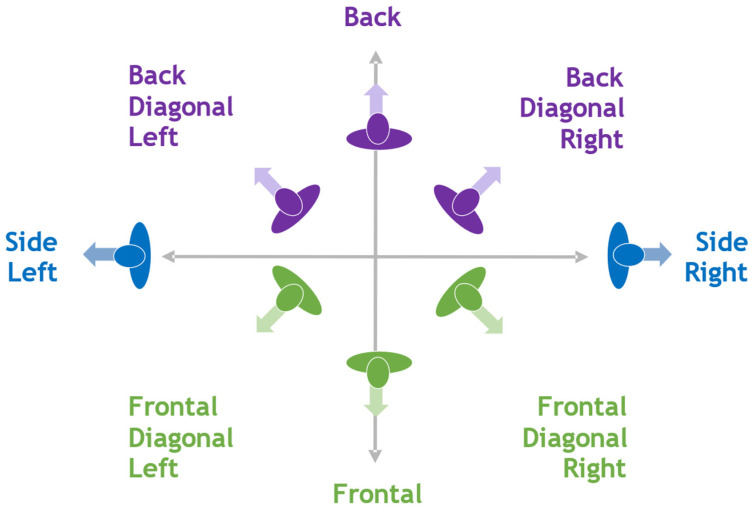
Body orientation categories.

**Figure 8:**
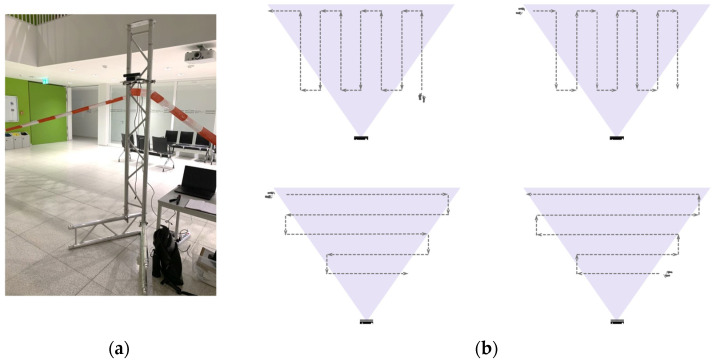
Experiment set up for all devices: in (**a**) the experiment arrangement; in (**b**) the different walking patterns with the start point and the camera’s position and field of view.

Experiment members are asked to walk in the isolated area in a combined walking pattern and body orientation in front of the camera for one minute per trajectory. For each body orientation representing back, frontal, diagonal, and side orientations, as detailed in Figure 7, four walking patterns should be completed from bottom to top and left to the right direction, as indicated in Figure 8, completing approximately 15 m per trajectory.

We classify the calculated body orientation angle into eight categories in slices of 45° and define an acceptable angle range for each category as displayed in Table 3 to evaluate each calculated body angle. The classification algorithm assigns the category labels with a 100% correspondence to an orientation. The accepted angle range is used to evaluate the margin error in calculating the body angle to identify how much is deviated from the expected result.

### 4.5. Evaluation and Results

We assess the calculated body angles accuracy in three stages: first, we evaluate whether the automatically detected body orientation falls into the correct category (i.e., the body angle with which the participant walked the experiment). The second evaluation aims to shed light on the accuracy, i.e., how large is the error, particularly for those automatically detected body orientations that did not fall into the correct category. Finally, the third evaluation addresses the context of social interaction in which we assess if the automatically detected body orientation falls into the maximum range of 30° for sustaining social interaction. Due to the findings of outliers in the experiment, we apply an interpolation correction by analysing the socially acceptable angle range and the nature of the outlier, showing the corrected body orientation’s angle results. For this evaluation, we compare the Kinect v2 results from our previous work presented at the IPIN 2021 conference [2].

#### 4.5.1. Intended Body Orientation Category Range

The evaluation compares the computed body orientation angle against the acceptable range for the intended orientation defined in Table 3. Figure 8a,b shows each device’s corresponding precision and recall for the single configuration. For the Kinect v2, the precision and recall are 0.82, respectively, with back diagonal and side orientations as the least accurate orientations. For the Azure Kinect, the precision and recall are 0.87, respectively, with the back orientation as the weakest. Finally, the Zed 2i possesses precision and recall of 0.83, with back diagonal right, back, and frontal diagonal left orientations with the lowest accuracy.

**Figure 9:**
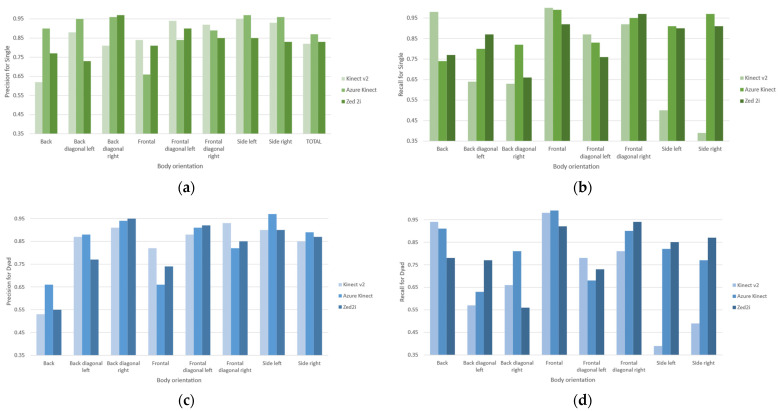
Precision and recall results for body orientation assessment for compiled measurements per device for single configuration in (**a**) and (**b**) and dyad configuration in (**c**) and (**d**), respectively.

For the dyad configuration, the precision and recall using the Kinect v2 are 0.79 and 0.80, where back diagonal and side categories have the lowest precision, as shown in Figure 9c,d. The Azure Kinect shows a general precision and recall of 0.81 up to 0.9 for frontal diagonal right, with frontal and back orientations precision below 0.70. The Zed 2i device orientations back, frontal, and back diagonal left show a low precision and recall lower than 0.77 with stronger orientations above 0.80 precision and recall up to 0.94. The most accurate orientations for the Kinect v2 benefit from the availability of the head rotation detection feature. In general, for the Azure Kinect and the Zed 2i, the back orientation is the most challenging orientation to detect, but despite not having the head/face rotation data available, the precision and accuracy results are higher than in the Kinect v2. For the Zed 2i, from the video review, we identified difficulties in detecting both participants, as one person was missing at a time, especially near the borders from the field of view.

#### 4.5.2. Intended Orientation Angle Deviation

For each category label, Table 4 and Table 5 show the angle deviation in degrees regarding the intended orientation in which the participants recreated the walking patterns. In general, for all three devices, for body orientations parallel to the depth camera, the average error is low for single and dyad, rising in diagonal body orientation categories. The most significant average error for the Kinect v2 and Zed 2i is the side orientations due to their orthogonality and the back orientation for the Azure Kinect. For the Kinect v2, the side-left and side-right high error results show difficulty collecting accurate skeleton data when the bodies possess a side orientation concerning the sensor’s position. The standard deviation in the single and dyad configurations does not surpass all devices’ next neighbouring category label. Exceptions are for the single configuration, the side-left and side-right orientations for Kinect v2 and Zed 2i with an error up to 1.5 adjacent classes. In the dyad configuration, the side-right orientation for the Kinect v2 deviates 1.5 adjacent classes. The back orientation deviates three adjacent classes for the Azure Kinect, resulting in the correct frontal orientation and two adjacent classes for the back diagonal right orientation.

**Figure 10:**
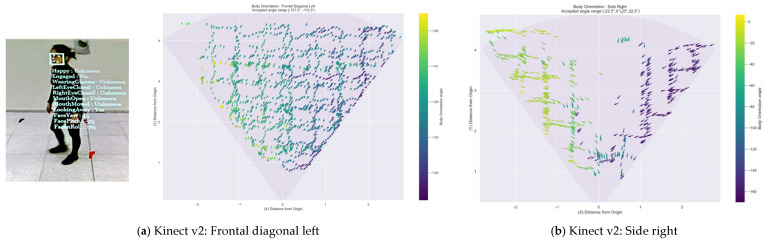

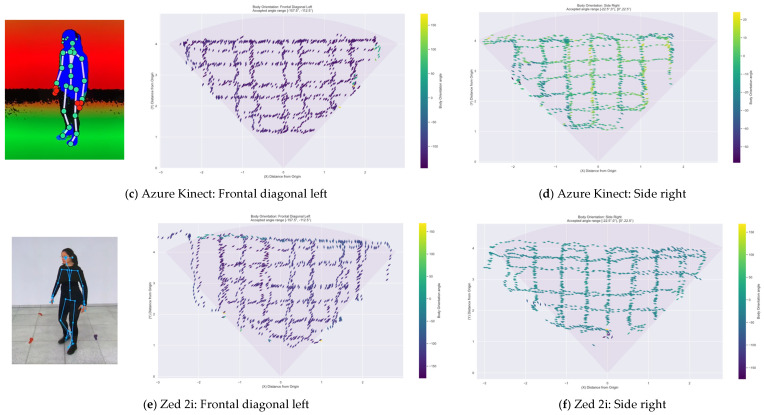
Calculated body orientation angles per sensor; (**a**,**c**,**e**) are highly accurate detected Frontal Diagonal orientation with participant view on the left; (**b**,**d**,**f**) show the detected Side Right orientation with the lowest accuracy for the Kinect v2.

The body orientation and the followed patterns are illustrated in Figure 10 for a highly accurate detected body orientation and a low accurate one for all three devices. The Kinect v2 depth camera extracts the head/face rotation and skeleton joints data, and the body disappears once it crosses the camera’s centre, recovering the body in a flipped orientation shortly after, as illustrated in Figure 10b. The Azure Kinect shows the designated patterns until the bodies reach the camera’s field of view limits in Figure 10c,d. For the Zed 2i, as it has a larger field of view than the other devices, is it possible to continue tracking the participants with difficulties in closer measurements and drawing the distance between each line inconsistently. 

#### 4.5.3. Intended Orientation inside the Interactional Space

**Figure 11:**
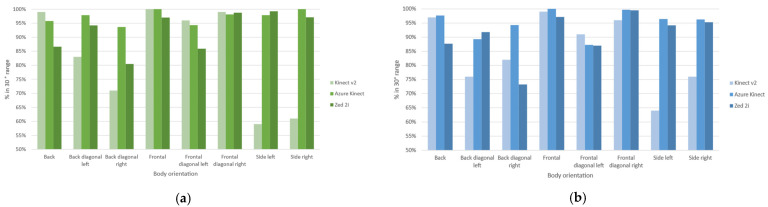
Achieved percentage for acceptable social interaction angle in range per device for single configuration in (**a**) and dyad configuration in (**b**).

Because we need the field-of-view extension, we evaluate the body orientation angle to assess the group’s focus of attention during an interaction. From the extension of the body orientation, we can define the focus of attention of each participant, and the intersection suggests a shared object of interest. In our case, we extend the participant’s field of view to the sides, drawing a 30° cone, and Figure 11 shows the results for the calculated angles classification within the interactional range. The interactional angle is detected around 80% of the time for most categories in all devices for single and dyad configurations. The socially acceptable orientation availability for the Kinect v2 in single configurations is low for side and back diagonal orientations due to the absence of joints and self-occlusion, improving in the dyad configurations by almost 10%. The Azure Kinect has the highest availability, with a socially acceptable range from 94% in the single configuration and 87% in the dyad configuration, up to 100% in both scenarios. The Zed 2i have comparable results to the Azure Kinect, ranging from 85% availability in single configurations and 73% in dyad configurations, with weaknesses in back diagonal right orientations for both configurations.

#### 4.5.4. Interpolation Correction

We identified outliers in each category during the classification of the body orientation angle. For this reason, to understand better whether inaccurate measurements occur systematically or whether they occur sporadically as isolated outliers, we consider the temporal dimension. In the latter case, we can correct erroneous measurements by considering the prior and subsequent measurements by smoothing out the error. We categorise these outliers into Neighbour outliers and Extreme outliers. Neighbour outliers are continuously wrong predicted angles along the walked trajectory. Extreme outliers are out of the median values with no temporal or spatial reason to appear. We use the recorded videos to examine both situations, review the intended body orientation angle and find an explanation for the wrong calculation. We apply an interpolation median correction to those spatial–temporal continuous values within 400 milliseconds with adjacent properly calculated values, and no external intervention is identified for the outlier to arise. We found that certain outliers followed a spatial–temporal pattern by plotting their location in the corresponding coordinate system. Afterwards, we inspected the video walking trajectories one-by-one to search for factors that might have led to the wrong skeleton-joints data extraction, related to the body’s relative position to the camera’s field of view, fluctuations in the body orientation while walking, and environment lighting changes.

**Figure 12:**
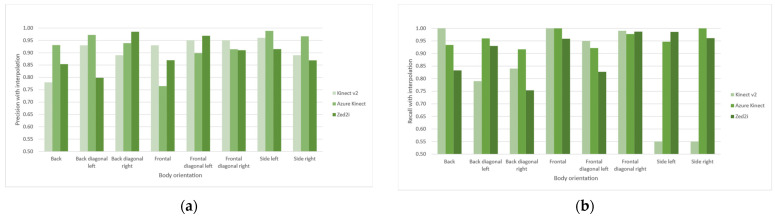

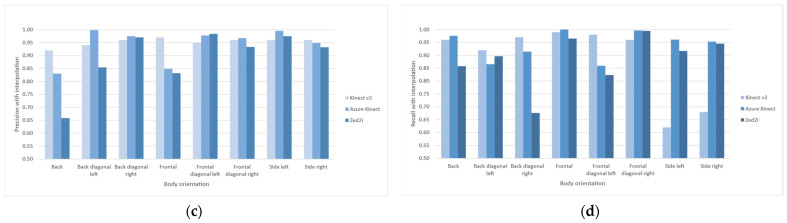
Precision and recall values for single configuration in (**a**,**b**), respectively; precision and recall values for dyad configuration in (**c**,**d**), respectively. Both after temporal interpolation.

**Figure 13:**
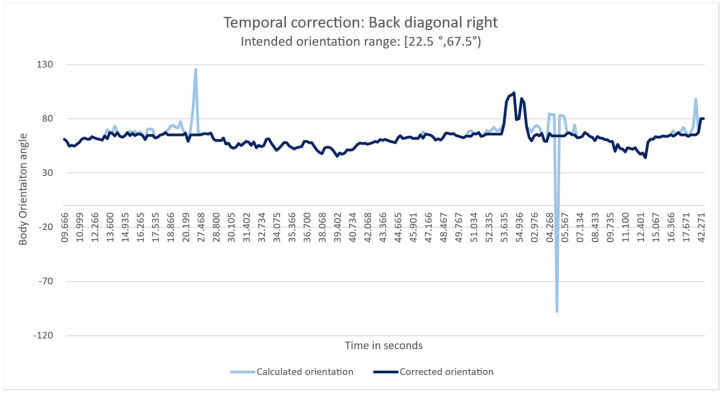
Temporal interpolation correction for the Back Diagonal Right orientation. In light blue, calculated body orientation angle with outliers. In dark blue, the corrected orientation angle.

We identified six distinct causes for an outlier: body entering the scene, body realignment, body proximity to the camera’s field of view limits, body with high proximity relative to the camera, depth range limit of camera’s field of view, and camera’s field of view centre. Body realignment is the natural movement as it moves to the desired location, which creates a forward movement from one shoulder to another as we step on foot at a time. The body entering the scene reveals that the device requires adjusting to the orientation. On the other hand, other outliers expose the weakest areas of the sensors’ field of view. We apply a temporal interpolation correction to manage these findings by taking the median value of two temporal and spatially pre and post continuous sample values. We then re-classify the calculated body orientation angles into the corresponding categories, obtaining the results described in Figure 12 with a visual representation of the correction shown in Figure 13. The new corrected body orientation angle values align with the accepted range angle for orientation. For single configurations, precision and recall values increase by 9% for the Kinect v2, 4% and 9% correspondently for the Azure Kinect, and 6% for the Zed 2i.

### 4.6. Discussion

The extraction of body orientation angles using skeleton data solely from depth cameras shows high accuracy and availability with the integration of spatial–temporal attributes to understand the human body’s mobility. Furthermore, there is evidence of the potential of depth sensor cameras to assess diverse body orientations by evaluating the calculated body angles against a set of categories in different walking patterns.

The Kinect v2 is mainly suitable for orientations aligned forward to the camera due to the algorithm training implemented in the device, which was trained primarily for these orientations for playing along with a console. However, it is feasible to have beneficial results for non-frontal orientations with the extracted skeleton joints and the head/face rotation data. The weakness relies on the body’s orthogonal orientation to the camera for side orientations, splitting the calculated orientation into two distinct areas. For the side orientation, the head/face rotation and upper skeleton joints are detected differently for each half of the trajectory, reflected in the value of 40% to 50% predicted accuracy.

The Azure Kinect has an accuracy greater than 90% in most orientations for single and dyad configurations, with a weakness in distinguishing between frontal or back orientation, which can be corrected by adding head/face rotation information. During the experiments, it was noticeable that if the body enters the scene from one of the borders, as shown in Figure 10c (top-right) and Figure 10d (bottom-left), the device takes time to adjust the proper orientation, especially in those backwards, recovering rather quickly, in around a second.

The Zed 2i in the single configuration highlights a high accuracy, between 80% to 95% for most orientations, with difficulties in diagonal orientations. As identified with the Azure Kinect, it needs time to adjust the skeleton once the body enters the scene from the borders, but as shown in Figure 10e,f, more spatial information can be recovered with the broad field of view. One primary concern is missing bodies in the scene at times, especially when they come back from leaving the camera’s field of view.

Notably, the side orientation was affected by the camera’s alignment of the body siding, misclassifying the calculated angle to neighbourhood categories. Concerning the temporal interpolation correction, body orientation angles can be misclassified due to self-occlusion and the delay of the camera algorithm to correct the body orientation, especially in the edges of the field of view, showing the relevance of spatial–temporal data analysis to identify anomalies in the experiment expected pattern.

Lastly, there is an overall precision and recall improvement of 16% for Kinect v2, 13% for Azure Kinect, and 9% for Zed 2i for the dyad configuration, in the lowest category classification: back diagonal and side orientations. The enhancement of more than 10% in the case of the Kinect v2 and the Azure Kinect suggests the possibility of increasing the accuracy of the body orientations by using spatial–temporal information during the movement of several subjects linked to the group’s temporality and habitation of a larger area. The positive results, particularly of the Azure Kinect in the interactional range evaluation, with single and multiple body detections, evidence the depth sensor cameras’ capability in generating social signals from skeleton joints datasets.

## 5. Evaluation in the Context of F-Formations

The following section describes the experimental setup for four social arrangements to detect F-Formations and the evaluation results, displaying the system’s potential in using measurements from the physically occupied space to interpret the socially occupied space.

### 5.1. Experiment Setup

This experiment follows the same setup regarding the delimited area, sensor location, data collection software, and configuration from the previous section. However, the task was different. Instead of walking along a path, participants were asked to stand at specific meeting points. Three meeting points are arranged in the free floor area in the camera’s field of view.

### 5.2. Definition of Encounter

**Figure 14:**
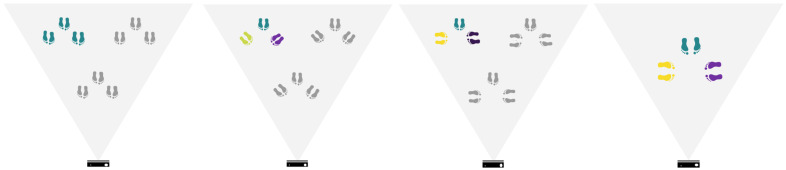
Description of the encounter locations with intended body orientations. From left to right: all-frontal, frontal-diagonal, frontal-vis a vis configuration (colour codes correspond to the Body Orientation angle bar in Figure 7).

The intended positions are marked to form a triangular pattern with vertices separated approximately 1.2 m. Participants stay on each one of the meeting points for 20 s per encounter, with frontal, side, and frontal diagonal orientations for all-frontal, frontal-diagonal and frontal-vis a vis interaction. The pattern is repeated in three distinct positions related to the cameras’ field of view and a single static position, as described in Figure 14.

### 5.3. Participants’ Description

For studying small gatherings in social encounters, two groups, one with two females and one male, the other one with two males and one female, with heights between 1.73 m and 1.81 m, met at different encounter points with a combination of body orientation and location. Each group concluded the task by producing thirty samples per device.

### 5.4. Evaluation Metrics and Results

The detection of groups is evaluated by comparing the number of stops identified by the algorithm against the intended meeting point. Additionally, we display the field of view of each participant during interaction to review how they intersect. In general, with the data collected with each depth camera, the algorithm identified groups by analysing the spatial–temporal interactional area’s attributes and location with more than 90% for the Kinect v2, and 100% for the Azure Kinect and the Zed 2i. Details on the number of bodies, stops and groups detected per meeting configuration are described in Table 6. For the Kinect v2, in the case of frontal-diagonal orientations, one-stop could not be detected due to the lack of data in assessing the orientation, which is explained by the orientation’s evaluation results. The Azure Kinect did not assign a unique identifier to participants, but the algorithm was able to discriminate them all and their stops thanks to the skeleton data’s high spatial–temporal resolution. On the other hand, the Zed 2i deviation for the frontal orientation is more evident in the group interactions and an additional body in the scene in 2 of 8 group interactions. However, similar to the Azure Kinect, the skeleton data resolution is high, allowing the algorithm to identify the authentic participants.

### 5.5. Discussion

**Figure 15:**
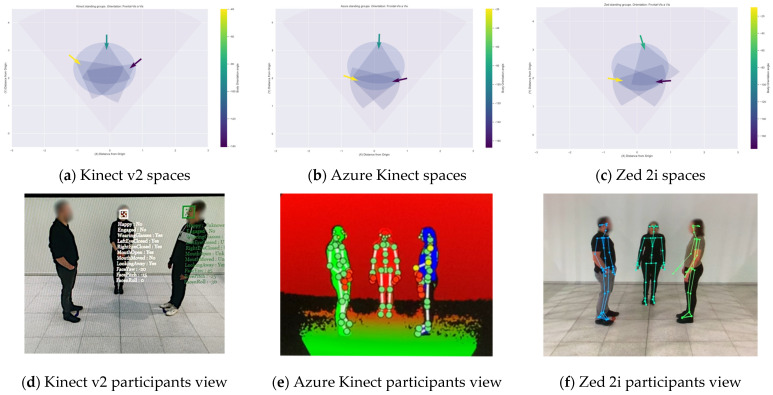
Group meeting points with frontal-vis a vis orientation per device (**a**) Kinect v2, (**b**) Azure Kinect, and (**c**) Zed 2i. Each colour differentiates a person participating in the group with their corresponding orientation angle. From (**d**–**f**), participants view per device.

With the skeleton data collected per depth camera, the group detection algorithm detected group members’ stops and membership in most designated meeting points. The field of view extension is calculated from the location and body orientation. Their intersection suggests the investigated focus of attention displayed in Figure 15 with a first approximation of the O-Space. The accuracy in detecting body orientations increases with differences per device for orientations facing the camera.

For the Kinect v2, in the diagonal orientations, a group is incomplete due to one member’s unavailability of its skeleton data, as expected for back-diagonal orientation and side orientations result from the classification evaluation. We conclude that the group members are more complicated to detect when the body orientation is sided with the depth camera, restricting the extraction of the skeleton joints. Moreover, for diagonal orientations, we acknowledge body occlusions between participants during trajectories when they move from one meeting point to another while analysing the video recordings, limiting the body angle measurement due to the absence of skeleton joints. On the other hand, despite these limitations and the lack of individual body identification, the Azure Kinect camera produced high spatial–temporal resolution skeleton data facilitating the algorithm detection of all trajectories and stops from the group members as it predicts with a lower level of confidence the occluded areas from other joint data. For each group member, it is possible to see the movement of the upper limbs for resolutions up to 5 cm, as seen in the control video recordings where the participant was moving in a circular motion in a single location. Finally, the Zed 2i possesses a high spatio-temporal resolution and an excellent overall identification of individuals during trajectories, although leaving the scene can leave to missing the body for more extended periods than the Azure Kinect.

**Figure 16:**
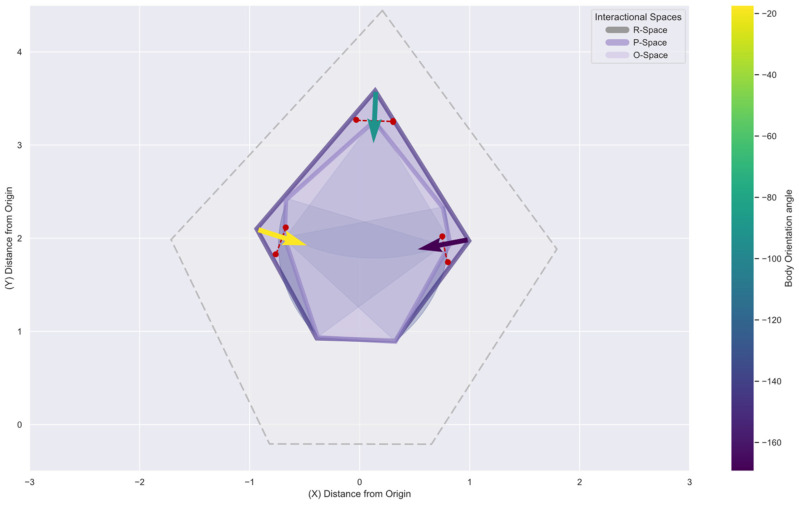
F-Formation’s interactional spaces view each body’s shoulder line (in red) with the detected orientation (arrows).

With the information derived from the skeleton data, it is possible to identify the different interactional spaces from an F-Formation, displayed in Figure 16. The participant’s field of view bounds the O-space, the attention focus. In combination with the bodies’ location, the field of view indicates the limits of the P-Space, where the participants sustain the interaction. Finally, the R-Space is constructed with a buffer determined by the social space [13], outside the inner interaction as a transactional space for the arrangement and disarrangement of the group, i.e., people leaving, arriving or standing at the socially occupied space.

By limiting the areas that shape an F-Formation with the physically occupied space, we can observe that the socially occupied space is stiff for tight body angles. With restricted access and more open orientations, the group interaction expands, granting clear access to external participants. This indicates that the interactional space components do not follow a standard shape, and it is related to the body orientation and proximity factors in the interaction.

## 6. Conclusions

This study performed a series of experiments to measure three different depth sensor cameras’ accuracy for assessing body orientation angles and purpose them to detect socially occupied spaces using the F-Formations model. First, we generated three datasets by walking in a combination of four trajectory patterns in eight body orientations in a single and dyad configuration. We observed that the Kinect v2 depth sensor’s accuracy is good in frontal, back, and diagonal orientations but weak when the user is aligned orthogonal to the camera in the case of side orientations. For the Azure Kinect, the depth sensor accuracy is higher in most orientations, with difficulties distinguishing frontal from back orientations as it lacks head/face rotation information. The Zed 2i, with its wide range, can collect more information, but it can omit bodies re-entering the scene. For other scenarios, the accuracy for the case of a strict categorisation proves to be 90%, 96%, and 89% for the Kinect v2, Azure Kinect, and Zed 2i, respectively, with a maximum standard deviation of 1.5, 3.0, and 1.5 angle classes. Finally, after the temporal interpolation correction for the socially acceptable interaction, the availability increases to 92.4%, 100.0%, and 99.8% for the single configuration and 94.9%, 100.0%, and 99.8% for the dyad configuration for the Kinect v2, Azure Kinect and Zed 2i, respectively.

Through this system, we can differentiate the components of the socially occupied spaces. For each device skeleton dataset, we reached 90% accuracy for the Kinect v2 and 100% for the Azure Kinect and the Zed 2i. The reached accuracy and socially acceptable angle availability from the Azure Kinect are adequate to detect F-Formations. Additionally, it does not depend on additional software to integrate head/face rotation data to improve the right-left correspondence, as in the case of Kinect v2 or RGB videos in the case of Zed 2i. Regarding our first approximation in detecting F-Formations’ interactional spaces, the algorithm identified the group members’ positions and assessed each participant’s field of view during an interaction. The interactional spaces could be delimited given the participant’s position, the study of proxemics, and the body orientation to assess the focus of attention.

Regarding resources and easiness in implementing the system, the hardware for using specific models can limit the performance, especially for the most recent devices, as it requires demanding resources. Nevertheless, the possibility of purchasing the technology is more significant than those specialised body tracking devices. For the particular use case of analysing group behaviour, depth cameras analyse individuals while making their identities more difficult to reveal, whereas the stereoscopic camera requires analysing raw video. Secondly, to collect data, the Zed 2i requires minimum light to discriminate details in the scene. We noticed this necessity in the experiment environment when the lights were dimmed, and the accuracy started to suffer, for which we sustained a proper illumination.

For the upcoming work, we aim to improve the socially occupied spaces detection algorithm and implement it in a desktop application to have live results for experimental group analysis. Improving the algorithm requires integrating parameters to appropriately limit the different interactional areas of the F-Formation model, the O-space, the P-Space, and the R-space from a spatial–temporal perspective and implementing and evaluation against other methods in the literature related to the assessment of social spaces in computer vision. Additionally, we intend to add more information regarding the dynamics of encounters by evaluating factors such as joining, leaving, or avoiding the group to facilitate the automatisation of human behaviour analysis. Lastly, integrating multiple sensors in one synchronised system to improve occlusion is also on the agenda as it may prove helpful in treating occlusions from the participants’ bodies and the loss of spatial–temporal data by integrating multiple points of view from the scene.

## Figures and Tables

**Table 1:** Depth cameras model availability with and without integrated skeleton tracking.

Device	Technique	Range	Skeleton Tracking
Azure Kinect	TOF	0.25–5.46 m	Yes, included
Kinect v2	TOF	0.50–4.50 m	Yes, included
pmd CarmBoard pico monstar	TOF	0.50–6.00 m	No
Intel Realsense D435i	Stereovision	0.30–3.00 m	Yes, to pay for
Intel Realsense D455	Stereovision	0.60–8.00 m	Yes, to pay for
Stereolabs Zed 2i	Stereovision	0.20–20.0 m	Yes, included
Orbbec Astra	Structured Light	0.60–8.00 m	Yes, to pay for
Orbbec Astra Pro Persee	Structured Light	0.40–8.00 m	Yes, to pay for

**Table 2:** Detailed technical comparison of the selected depth cameras.

	Kinect v2	Azure Kinect	Zed 2i
Year	2016	2020	2020
Technology	TOF	TOF	Stereovision
Colour camera resolution	1920 × 1080 px @30 fps	4096 × 3072 @30 fps	2× (2208 × 1242) @15 fps 2× (1920 × 1080) @30 fps 2× (1280 × 720) @60 fps
Depth camera resolution	512 × 424 px @30 fps	Narrow: 654 × 576 @30 fps Wide: 1024 × 1024 @30 fps	
Field of view	70° H–60° V	Narrow: 75° H–65° V Wide: 120° H–129° V	110° H–70° V
Depth extent	0.5 m–4.5 m	0.25 m–5.46 m	0.2 m–20 m
Coding language	C#	C, C#	C, C++, Python
Skeleton joints	25	32	34

**Table 3:** Description of the body orientations label with the intended and defined acceptable angle range.

Body Orientation Label	Intended Orientation Angle	Accepted Angle Range
Side right	0°	[−22.5°, 0°), [0°, 22.5°)
Back diagonal right	45°	[22.5°, 67.5°)
Back	90°	[67.5°, 112.5°)
Back diagonal left	135°	[112.5°, 157.5°)
Side left	−180°/180°	[157.5°, 180°), (−180°, 157.5°]
Frontal diagonal left	−135°	[−157.5°, −112.5°)
Frontal	−90°	[−112.5°, −67.5°)
Frontal diagonal right	−45°	[−67.5°, −22.5°)
Back diagonal left	0°	[−22.5°, 0°), [0°, 22.5°)

**Table 4:** Single Configuration: Evaluation of body intended orientation angle deviation (IOD) intended orientation angle (IO) in degrees. Bold numbers indicate the highest values.

Body Orientation	Kinect v2		Azure Kinect		Zed 2i	
	IOD AVG	IOD STD	IOD AVG	IOD STD	IOD AVG	IOD STD
Back	6.85	6.51	**30.51**	**58.84**	18.18	**24.12**
Back diagonal left	19.54	11.81	15.72	**29.60**	14.64	22.70
Back diagonal right	21.74	15.82	22.73	**43.11**	21.71	21.45
Frontal	4.81	4.12	5.51	5.01	10.27	7.66
Frontal diagonal left	13.61	8.83	15.20	13.57	18.69	22.32
Frontal diagonal right	11.98	6.95	11.06	16.76	9.45	10.68
Side left	**34.81**	**34.46**	19.78	13.67	**35.29**	10.65
Side right	**35.62**	**31.55**	8.19	6.30	12.38	10.97

**Table 5:** Dyad Configuration: Evaluation of body intended orientation angle deviation (IOD) against intended orientation angle (IO) in degrees. Bold numbers indicate the highest values.

Body Orientation	Kinect v2		Azure Kinect		Zed 2i	
	IOD AVG	IOD STD	IOD AVG	IOD STD	IOD AVG	IOD STD
Back	8.37	8.37	13.39	**26.62**	15.883	17.499
Back diagonal left	**22.96**	**22.96**	19.53	16.08	15.975	14.067
Back diagonal right	20.35	20.35	14.53	20.39	23.730	18.311
Frontal	6.53	6.53	6.50	5.26	9.690	9.121
Frontal diagonal left	14.8	14.8	17.47	12.84	17.610	19.293
Frontal diagonal right	13.84	13.84	11.58	7.35	9.558	7.610
Side left	**28.1**	**28.1**	**23.08**	18.97	**26.411**	**24.267**
Side right	**36.93**	**36.93**	15.53	10.45	15.005	22.984

**Table 6:** Group detection results with the number of bodies and stops per configuration. Bold numbers indicate a lower or higher number of bodies detected.

Stops	Orientation	Kinect v2			Azure Kinect			Zed 2i		
		Bodies	Stops	Groups	Bodies	Stops	Groups	Bodies	Stops	Groups
3	Frontal	3	9	3	3	9	3	**4**	9	3
3	Frontal/Face to face	3	9	3	3	9	3	3	9	3
3	Frontal/Diagonal	**2**	8	2	3	9	3	3	9	3
1	Frontal	3	9	1	3	3	1	3	1	1
1	Frontal/Face to face	3	9	1	3	3	1	**4**	1	1
1	Frontal/Diagonal	3	8	1	3	3	1	3	1	1

## Data Availability

The data collected in these studies are openly available at https://osf.io/xhwgm/ (accessed on 15 March 2022). The tutorials and code to create a new interface for all devices can be found at https://github.com/violetasdev/bodytrackingdepth_course (accessed on 15 March 2022). For the Azure Kinect, we implement a modified version of k4.net, the final version is available at https://github.com/violetasdev/k4a.net (accessed on 15 March 2022). The Kinect v2 graphs can be reproduced at https://osf.io/ghz79/ (accessed on 15 March 2022).

## Abbreviations

The following abbreviations are used in this manuscript:

IO	Intended orientation angle
IOD	Intended orientation angle deviation
IR	Infrared
RGB-D	RGB depth
SDK	Software development kit
TOF	Time of Flight
